# Physical Activity, Residential Environment, and Nature Relatedness in Young Men—A Population-Based MOPO Study

**DOI:** 10.3390/ijerph15102322

**Published:** 2018-10-22

**Authors:** Soile Puhakka, Riitta Pyky, Tiina Lankila, Maarit Kangas, Jarmo Rusanen, Tiina M. Ikäheimo, Heli Koivumaa-Honkanen, Raija Korpelainen

**Affiliations:** 1 Department of Sports and Exercise Medicine, Oulu Deaconess Institute, P.O. Box 365, 90100 Oulu, Finland; riitta.pyky@odl.fi (R.P.); raija.korpelainen@odl.fi (R.K.); 2 Center for Life Course Health Research, Faculty of Medicine, University of Oulu, P.O. Box 5000, 90014 Oulu, Finland; tiina.lankila@oulu.fi; 3 The Geography Research Unit, Faculty of Science, University of Oulu, P.O. Box 3000, 90014 Oulu, Finland; jarmo.rusanen@oulu.fi; 4 Medical Research Center, Oulu University Hospital and University of Oulu, P.O. Box 5000, 90014 Oulu, Finland; maarit.kangas@oulu.fi (M.K.); tiina.ikaheimo@oulu.fi (T.M.I.); 5 Research Unit of Medical Imaging, Physics, and Technology, Faculty of Medicine, University of Oulu, P.O. Box 5000, 90014 Oulu, Finland; 6 Center for Environmental and Respiratory Health Research, University of Oulu, P.O. Box 5000, 90014 Oulu, Finland; 7 Department of Psychiatry, Kuopio University Hospital (KUH), P.O. BOX 100, 70029 Kuopio, Finland; heli.koivumaa@kuh.fi; 8 Department of Psychiatry, Lapland Hospital District, P.O. BOX 8041, 96101 Rovaniemi, Finland; 9 Department of Psychiatry, Institute of Clinical Medicine, University of Eastern Finland, P.O. BOX 1627, 70211 Kuopio, Finland

**Keywords:** nature relatedness, physical activity, physical activity with parents, residential environment, GIS

## Abstract

*Background*: In general, nature relatedness is positively associated with physical activity, health, and subjective well-being. However, increased residence in urban areas, and the decrease in natural spaces, may affect the younger generation most adversely. The associated environmental changes can increase youths’ risk of spending most of their time indoors, and weaken their nature relatedness, making them less likely to enjoy nature’s health benefits. This is a serious public health issue, since inadequate physical activity, combined with minimum time spent in green space, can affect health across the whole lifespan. Thus, to develop effective interventions for physical activation and promote health and well-being among young men, further knowledge of the determinants of their nature relatedness is necessary. *Aims*: To explore factors related to nature relatedness, including physical activity, physical activity with parents, and residential environment. *Methods*: The study population consisted of all 914 young men (mean—17.8 years; SD—0.5) who participated in mandatory call-ups for military service and completed the study questionnaire in 2013. The questionnaire inquired about their nature relatedness, demographic characteristics, socioeconomic status, physical activity, health, and subjective well-being. A geographic information system (GIS) was used to assess the features of their residential environments. Multivariable linear regression was used to analyze the data. *Results*: Physical activity (*p* = 0.021) and physical activity with parents at primary school age (*p* = 0.007), and currently (*p* = 0.001) as well as good self-rated health (*p* = 0.001), and father’s higher socioeconomic status (*p* = 0.041), were positively connected to nature relatedness. *Conclusions*: Physical activity in general, physical activity with parents, and nature relatedness were positively related. This knowledge can be utilized in promoting physical activity and health among young men.

## 1. Introduction

### 1.1. Background

There is growing evidence that exposure to nature has beneficial implications for physiological and psychological health. Both observational and experimental studies support the existence of a connection between green space and a range of health outcomes, including physical activity, perceived general health, and mental health and well-being [1,2,3,4,5]. The pathways connecting green space and health are likely to include improving air quality, physical activity, social cohesion, and stress reduction. These factors are probably highly interactive factors [6,7,8,9,10,11].

Naturally green areas and recreational areas in cities are decreasing in number and size, or non-existent [12]. In the United States, adolescents spend almost 60% of their waking hours sitting, and young people spend an increasing amount of their free time engaged in indoor activities, such as those involving the internet, social media, and games [13,14]. Worldwide, 80% of adolescents do not meet the current recommendations for adequate physical activity [15]. Such adverse habits will affect health across the whole lifespan [16]. Due to urbanization, the prevalence of stress-related diseases has also increased [17]. These phenomena could become a larger public health issue [18]. It is known that adolescents spend little time in green space [19]. Weakening nature relatedness among members of the younger generation makes them less likely to make use of nature’s health benefits. Thus, to improve young people’s relationship with nature, and the possibility that they will achieve the associated positive health effects, it is essential to understand the factors tied to their nature relatedness. Our hypothesis is that young men with stronger nature relatedness are physically more active, and their health is better compared to young men with weaker nature relatedness.

### 1.2. Nature Relatedness

Nature relatedness includes the affective, cognitive, and experimental aspects of the human–nature relationship [20]. According to Wilson’s “biophilia hypothesis”, there could be an instinctive bond between human beings and other living systems [21,22]. Ulrich’s psychoevolutionary theory is highly based on this hypothesis. It states that, in order to experience nature’s positive effects, one needs to have a good connection to it, and the ability to expose oneself to it [23]. Among adults, nature relatedness is connected to health and well-being [24,25]. The younger generation naturally has weaker nature relatedness than the older ones, whose members have been exposed to nature to a greater extent, and to technology to a lesser extent [12,26]. However, many factors can affect the strength of nature relatedness. For example, lack of access to nature (e.g., if it is a long distance from home) and urbanization may hinder the transfer of common cultural heritage (e.g., the link to animals and nature) from one generation to another. Thus, the gap between nature and people can lead to weakened nature relatedness. Even if concrete factors influence nature relatedness, people have individual ways of experiencing nature [27]. In addition, childhood and cultural heritage can have strong effects on thoughts and attitudes related to nature [28,29]. Thus, more information is needed to identify the determinants of nature relatedness in young men. Some studies suggest that people with stronger nature relatedness spend more time outdoors, compared to those whose nature relatedness is weaker.

The aim of the present study was to explore the factors associated with nature relatedness among young men, with special emphasis on physical activity (PA), green residential environments, and the parental role in PA.

## 2. Methods

This study is part of a comprehensive population-based study (MOPO). Its main purpose is to promote effective health and physical activity, and to prevent social marginalization among young, conscription-aged Finnish men [30]. The study took place in the City of Oulu in Northern Finland in 2013, during call-ups for military service. Military service or civic duty is compulsory for all Finnish male citizens, and the Finnish Defense Forces organize conscription every year. The entire age cohort of 18-year-old men, except those whose physical or mental health or psychological capacities do not allow independent living, participate in it. Thus, the call-ups provide a large, population-based representative sample of young men. The study was conducted in accordance with the Declaration of Helsinki of 1964, as revised in 2000, and the Ethical Committee of Northern Ostrobothnia Hospital District (ETMM123/2009) approved it. The subjects had the right to refuse to participate or to withdraw from the study. The manuscript does not contain any individual person data.

Availability of data and material: Data is available from the University of Oulu, NFBC, for research collaborators who meet the criteria for accessing confidential data. Please, contact project center for further information: NFBC projectcenter@oulu.fi.

### 2.1. Study Population

All the young men who attended the call-ups for military service in 2013 (*N* = 1352) were invited to the present study. Out of the entire age cohort group, 914 subjects (mean age—17.8; SD—0.5 years; 68% of the age cohort) agreed to complete the questionnaire concerning their nature relatedness, physical activity, physical activity with parents, psychological and physical health, life satisfaction, nutrition, smoking and alcohol intake, and socioeconomic factors, such as parent’s occupation. Weight, height, and body mass index (BMI) were measured.

### 2.2. Measures

Nature relatedness was assessed by focusing on 18 items concerning young men’s thoughts and experiences, and the benefits of nature (Table A1), which were based on the Nature Relatedness Scale [31], the theory of motivations of visiting natural areas, and the work of Staats and Hartig (the theory of social aspect of being outdoors) [32,33]. The measure was calculated based on the scoring of these statements. Each statement had three response alternatives (1 = “always or mainly”, 2 = “sometimes”, and 3 = “seldom or never”), except for statement number 3 (“The thought of being deep in the woods, away from civilization, is frightening”), which had reversed scoring (Table A1). The range of the index was 0–36 (with a higher score indicating stronger contact with nature). The continuous variable was used in the multivariable linear regression. For the univariate analyses, the index was categorized into four quartiles (1 = Weak (score ≤ 14), 2 = Moderate (score of 15–18), 3 = Good (score of 19–26), and 4 = Strong (score ≥ 27)), and finally dichotomized by combining categories 1–2 (0 = “Lower half of the group”) and 3–4 (1 = “Upper half of the group”).

Physical activity was measured through the question, “How much do you exercise during your leisure time?”. The responses originally constituted a four-point scale: 1 = “I read, watch television, and do tasks that do not require physical activity or sweating”; 2 = “I walk, bicycle, or engage in some other kind physical activity for at least 4 hours per week”; 3 = “I exercise vigorously or do equivalent activities for at least 2 hours per week, on average”; 4 = “I exercise competitively several days a week”. The variable was transformed into a two-point scale (1 = “Low leisure time physical activity”; 2–4 = “Higher leisure time physical activity”). The participants were asked whether or not they exercised with their parents (1) during childhood (≤6 years); (2) at primary school age (7–12 years); (3) at secondary school age (13–15 years); (4) currently.

The participants were also asked about their current smoking habits and alcohol intake (“Do you smoke/use alcohol?”) and given two response alternatives (1 = “Yes” or 0 = “No”).

An open-ended question requested information about the parents’ socioeconomic statuses (SESs). The responses fit into six categories from the Classification 89 of Occupations 2010 by Statistics Finland (2013): 1 = “Managers and entrepreneurs”, 2 = “Professionals”, 3 = “Associate professionals”, 4 = “Service and sales workers”, 5 = “Manual workers”, and 6 = “Others (unemployed, pensioner, student, unknown)”. Categories 1–3 were combined to represent higher SESs; categories 4–5 were combined to represent lower SESs; category 6 (unknown and perished) was excluded.

Information regarding self-rated health was determined through the question, “How good is your self-rated health?” There were 5 response alternatives: 1 = “Good”, 2 = “Pretty good”, 3 = “Moderate”, 4 = “Pretty poor”, and 5 = “Poor”. The categories were dichotomized: categories 3–5 (0 = “Poor”) and categories 1–2 (1 = “Good”). Low self-rated health has been shown to be associated with outcomes, such as higher mortality in several population-based studies [34].

Life satisfaction was assessed using a 4-item scale on happiness, interest in life, ease of living, and loneliness (range: 4–20). Scores were based on the following responses: 1 = “Very interesting/happy/easy/not at all lonely”, 2 = “Fairly interesting/happy/easy”, 3 = “Cannot say”, 4 = “Fairly boring/unhappy/hard/lonely”, and 5 = “Very boring/unhappy/hard/lonely” [35].

### 2.3. Geographic Information Systems Methods

Geographic information systems (GIS) methods enabled the investigation of the relationship between environmental factors and nature relatedness. The GIS methods required the collection, assimilation, and preprocessing of digital datasets. The spatial information in this study was linked to the exact locations of the participants’ residences, based on geographical coordinates. The ESRI GIS-tool, ArcMap 10.2 [36], was used to characterize participants’ residential environments by creating a variable 500 m-to-5 km specific buffer zone around each individual’s residence (coordinate points, as the crow flies). These size buffers have been used in similar studies involving everyday environments [37,38] and nearby nature, which has been critical for providing health benefits for subjective well-being [39].

The tool was also utilized to calculate the composition of land cover (land use) within the buffer and to create a variable, which represented the amount of green space in the participants’ residential areas. Previously, land cover data has been used in similar studies [38,40]. The data was based on Corine land cover data (one grid: 20 m × 20 m), which entailed spatial information regarding land cover obtained from satellite data [41]. The calculation of the variable involved 5 main levels: 1 = “Artificial surfaces”, 2 = “Agricultural areas”, 3 = “Forests and semi-natural areas”, 4 = “Wetlands”, and 5 = “Water bodies”. They were categorized as (1) built environment (Level 1) and (2) natural residential environment (Levels 2–5). Corine land cover data was also modified to measure the number of natural elements in residential environment each participant’s residential environment (for instance, forests; transitional woodlands/shrub; wetlands and peat bogs; water bodies, such as rivers, lakes and seas, salt marshes, inland marshes; bare rocks; beaches; dunes and sand planes; non-irrigated arable land; and natural grassland). In addition, we calculated the number of natural elements within a 5 km radius of each participant’s residence. The supposition was that, the greater the number of natural elements in a residential environment, the more diverse it was. Distance to the closest park (in meters) was defined from Corine by identifying the shortest distance between two objects (as the crow flies). In this case, it was the distance between the coordinates of each participant’s residence and the closest park or forest.

### 2.4. Statistical Analysis

The variables were tested for normality. The statistical significance of the difference between the study groups was first analyzed using cross-tabulation and the chi-square test for the categorical variables, and Pearson’s correlation test for the continuous variables.

Multiple linear regression analyses were used to reveal the factors associated with nature relatedness in the total population, and among those with weaker and stronger nature relationships. The explanatory variables were tested for multicollinearity. The highest two-tailed correlation allowed was 0.6. The variance inflation factor (VIF) value was supposed to remain below 10, and the tolerance measure above 20. In the second step, influential observation was handled using Cook’s distance, meaning that observations significantly above 1 were influential, and had to be excluded. Missing data was excluded through the pairwise deletion of cases. The “stepwise” method was used in the analysis. In addition, we used physical activity as a dependent variable in the logistic regression analysis, which was performed to explore the connection between physical activity, nature relatedness, and other variables mentioned in this study.

The residuals were checked for normal distribution. Moreover, the data was analyzed using PASW Statistics software [42].

## 3. Results

Altogether, 914 young men, who participated in conscription in 2013, completed the questionnaire. In addition, 430 of them (47.0%) were classified as participants with stronger nature relatedness (score ≥ 19), and 484 as participants with weaker nature relatedness (score < 19) (Table 1).

The young men with stronger nature relatedness had better self-rated health, had lower alcohol intake, smoked less, were physically more active, spent more time in nature, and had had more PA.

In multivariate linear regression (Table 2) involving the total population group, the following variables were positively related to nature relatedness: physical activity (*β* = 2.13, CI (confidence interval) = 0.31, 3.94), primary school-age physical activity with parents (*β* = 2.06, CI = 0.57, 3.56) and current physical activity with parents (*β* = 2.88, CI = 1.12, 4.65), good self-rated health (*β* = 2.84, CI = 1.15, 4.53), and father’s higher SES (*β* = 1.38, CI = 0.05, 2.07). All of the five variables were significantly associated with nature relatedness (*p* < 0.050). This five-variable linear regression model accounted for 9.1% (*R* squared) of the variance in the nature relatedness.

When those with weaker natural relatedness were studied separately, the following variables were found to be positively associated with nature relatedness: spending time in nature (*β* = 3.37, CI = 2.20, 4.53), primary school-age physical activity with parents (*β* = 1.68, CI = 0.48, 2.89), and father’s higher SES (*β* = 1.19, CI = 0.10, 2.31) (Table 3).

In the secondary analysis of this study, we used physical activity as a dependent variable. The results revealed that self-rated health (OR (Odds ratio) = 0.17, *p* ≤ 0.001, CI = 0.120, 0.252) and nature relatedness (OR = 1.04, *p* ≤ 0.001, CI = 1.021, 1.064) were positively associated with physical activity among young men.

## 4. Discussion

The purpose of this this cross-sectional population-based study was to identify factors associated with nature relatedness among adolescent men, with special reference to physical activity, the residential environment, and subjective well-being. We found that better self-rated heath, a healthier lifestyle, and more time spent in nature, were related to nature relatedness among young men. Furthermore, physical activity, in general, and physical activity with parents (primary school-age and currently), as well as the father’s higher SES, were positively associated with nature relatedness.

Our special study was interested in the association between nature relatedness and physical activity. It is known that people with stronger nature relatedness tend to prefer—that is, make more frequent visits to—naturally green areas and parks [31]. In addition, environmental greenness has been found to be related to physical activity in several studies but, thus far, mainly among adults [43,44,45,46]. Our study suggested that spending time in nature was positively connected to nature relatedness, especially among those whose nature relatedness was weaker.

Previously, only a few studies focused on the roles of nature relatedness and physical activity. Based on previous studies on adults, it is possible that greenness and the positive effects of nature could also play a key role in activating young men. There is some evidence that exercising outdoors requires less exertion than exercising indoors [47,48]. Thus, young men with stronger nature relatedness might prefer to exercise outdoors. Furthermore, it has been reported that, for those with stronger nature relatedness, outdoor exercise has more benefits for well-being than exercising indoors [49,50,51]. Still, it is important to bear in mind that nature relatedness entails more than visiting green areas and exercising outdoors. It has been suggested that one must subjectively “perceive” nature, especially greenness, to obtain its health benefits [39,52]. Thus, further and more comprehensive research is needed to examine the relationship between nature relatedness and physical activity. We also need to identify the natural environments that can strengthen young people’s nature relatedness, in order to improve their physical activity and subjective well-being [53].

In the present study, we found that an association between nature relatedness and physical activity with parents was also evident. In the ideal case, physical activity experienced with parents in childhood resulted in time spent together outdoors. Among young men with weaker nature relatedness, physical activity with parents at primary school age was positively connected with nature relatedness. Frequent contact with nature in childhood might have led to long-lasting and strong nature relatedness, but that was also a cultural phenomenon [54]. Louv [55] suggested that modern parents might not allow their children to explore nature, due to their own prejudices and lack of knowledge. Nature has been suggested to be rooted in human biology, but nature experiences also play a key role in the adoption of nature as a part of one’s life [12]. Finland is a sparsely populated country, which means that nature is always near. When Finnish families exercise together, they typically go outdoors to play or for a forest trip. Thus, we can at least suggest that physical activity with parents, in the present study, consisted mainly of outdoor activities. In addition, early learned motivation to exercise might have encouraged contact with nature. Our study also showed that those young men who engaged in physical activity with their parents in early childhood were more likely to maintain these activities with their parents in adolescence. However, longitudinal studies are necessary to examine how parents can positively affect their children’s nature relatedness and the possibilities of their experiencing nature’s positive effects.

According to our results, self-rated health was positively connected to nature relatedness. Furthermore, in a previous comparable study, an association between green residential environment and perceived health was observed. It is most likely that self-rated health is strongly associated with the other indicators of well-being, such as physical activity [24]. Young men with stronger nature relatedness are also more likely to be exposed to nature than those with weaker nature relatedness. Thus, the former have more of an opportunity to experience nature’s positive effects. The combination of all these indicators of well-being suggests that young men’s vitality improves when their nature relatedness is stronger [56].

The combination of the qualitative and quantitative data, used in the present study, can be applied to the study of nature relatedness in other age groups as well. The information produced can be used for health interventions among young men, particularly, nature-related interventions for PA.

Strengths and limitations: The major strength of this study was its population-based setting. Another advantage was the high compliance with the questionnaire. Also, geographic information systems methods, based on exact geographical coordinates, were used for the objective quantitative assessment of the features of the participants’ residential environments. The use of questionnaire information to assess physical activity, general health, and nature relatedness, may have involved some information bias. Physical activity was assessed through a questionnaire, and exact data on the outdoor physical activity was not available. Our study was also limited because of its cross-sectional setting. Reliance on self-reported measures of behavioral factors, such as physical activity, is a limitation in surveys of this kind. Social desirability bias can lead to overreporting of physical activity [57,58] and overestimation of higher intensity (i.e., vigorous) physical activities than in the low-to-moderate levels [59]. Such an obviously non-differential bias is, however, unlikely to affect the association between nature relationship and the explanatory factors. Questionnaire methods do not generally provide accurate data on individual energy expenditure, but they are considered useful for grouping people into categories on the basis of their physical activity [60].

## 5. Conclusions

This study showed that physical activity, in general, physical activity with parents, and nature relatedness, were positively related. This knowledge can be utilized in promoting physical activity and health among young men.

## Figures and Tables

**Table 1:** Characteristics of the study population (*n* = 914) by nature relatedness (NR) *. Values are means (SD) if not otherwise stated.

Characteristics	All (*n* = 914)	NR Score ≥ 19 (*n* = 430)	NR Score ≤ 18 (*n* = 484)	*p* *
Age, years	17.8 (0.5)	17.7 (0.5)	17.8 (0.6)	0.678
Weight, kg	72.9 (6.4)	72.3 (13.5)	73.5 (14.7)	0.275
Height, cm	177.8 (14.1)	177.6 (6.2)	178.0 (6.2)	0.472
BMI, kg/m^2^	23.0 (4.2)	22.8 (3.9)	23.1 (4.5)	0.322
Good self-rated health, *n* (%)	660 (74.5)	343 (79.7)	317 (65.4)	<0.001
Life satisfaction (range: 4–20) ^a^	9.97 (2.0)	9.79 (2.0)	10.13 (2.1)	0.015
Current alcohol intake (yes), n (%)	572 (63.2)	236 (54.8)	336 (69.4)	<0.001
Current smoking (yes), *n* (%)	246 (27.2)	95 (22.0)	151 (31.1)	0.002
Physical activity ≥ 4 per week (yes), *n* (%)	722 (80.6)	369 (85.8)	353 (72.9)	<0.001
Physical activity with parents during childhood (yes)	672 (77.8)	340 (79.0)	332 (68.5)	<0.001
Physical activity with parents at primary school age (yes), *n* (%)	618 (70.7)	316 (73.4)	302 (62.3)	<0.001
Physical activity with parents at secondary school age (yes), *n* (%)	241 (27.9)	148 (34.4)	93 (19.2)	<0.001
Current physical activity with parents (yes), *n* (%)	148 (17.3)	93 (21.6)	55 (11.3)	<0.001
Mother’s higher SES, *n* (%)	406 (53.0)	193 (44.8)	213 (44.0)	0.942
Father’s higher SES, *n* (%)	367 (49.9)	194 (45.1)	173 (35.7)	0.032
Amount of natural space in residential environment (500 m buffer), km^2^	0.36 (0.15)	0.37 (0.15)	0.35 (0.14)	0.044
Distance to closest park, m	3744 (4241)	3764 (4834)	3166 (3616)	0.033
Distance to closest forest, m	455.60 (501.01)	453.10 (508.22)	457.82 (495.02)	0.887
Spends time in green areas (yes), *n* (%)	532 (59.0)	361 (40,183.9)	171 (19,035.3)	<0.001
Number of natural elements in residential environment	41.53 (10.6)	41.55 (10.4)	41.51 (9.9)	0.071
Nature relatedness	19.07 (8.9)	26.64 (4.4)	12.35 (5.9)	<0.000

* *p*-values (stronger nature relatedness vs. weaker nature relatedness group) independent samples crosstabs/chi-squared test or independent samples *t*-test. ^a^ Life satisfaction: higher score indicates lower life satisfaction. Numbers do not match due to missing values. NR score: ranging 0–36, higher score indicating better NR (lower half of the group ≤ 18 and higher half of the group ≥ 19).

**Table 2:** Factors associated with nature relatedness among young men (*n* = 914) according to multivariable linear regression.

Variable *	β	95% CI	*p*
Physical activity ≥ 4 h per week	2.13	0.31, 3.94	0.021
Physical activity with parents at primary school age	2.06	0.57, 3.56	0.007
Current physical activity with parents	2.88	1.12, 4.65	0.001
Good self-rated health	2.84	1.15, 4.53	0.001
Father’s higher SES	1.38	0.05, 2.70	0.041

* yes/no if not continuous

**Table 3:** Factors associated with nature relatedness among young men with weaker nature relatedness (NR score ≤ 18).

Variable *	*β*	95% CI	*p*
**Weaker nature relatedness**			
Spending time in nature	3.37	2.20, 4.53	<0.001
PA with parents at primary school age	1.68	0.48, 2.89	0.006
Father’s higher SES	1.19	0.00, 2.31	0.038

* yes/no if not continuous

## Abbreviations

PA	Physical activity
GIS	Geographic Information Systems
SES	Socioeconomic status

## Appendix

**Table A1:** Scoring of nature relatedness.

Statement	Always or Mainly	Sometimes	Seldom or Never
1. I spend time in natural areas (e.g. recreational grounds, nearby forests, parks, national parks, and other nature reserves and water bodies).	2	1	0
2. I enjoy being outdoors, even in unpleasant weather.	2	1	0
3. The thought of being deep in the woods, away from civilization, is frightening.	0	1	2
4. Experiencing nature is an important part of my well-being.	2	1	0
5. My thoughts become clearer.	2	1	0
6. Tomorrow looks brighter.	2	1	0
7. I get new energy and eagerness to engage in my daily tasks.	2	1	0
8. I relax and recuperate.	2	1	0
9. I get more self-confidence.	2	1	0
10. I get new, inspiring thoughts.	2	1	0
11. I enjoy meeting new people during my visits to nature.	2	1	0
12. I enjoy the company of the people closest to me.	2	1	0
13. It is easy to discuss your personal issues in nature.	2	1	0
14. I enjoy being alone.	2	1	0
15. I enjoy silence.	2	1	0
16. I can test my personal limits.	2	1	0
17. I feel that exercising outdoors improves my physical condition.	2	1	0
18. I feel my physical wellness improving.	2	1	0
